# Highly Rotationally Excited N_2_ Reveals Transition-State Character in the Thermal Decomposition of N_2_O on Pd(110)

**DOI:** 10.1021/jacs.3c01127

**Published:** 2023-05-24

**Authors:** Jiamei Quan, Rongrong Yin, Zibo Zhao, Ximei Yang, Alexander Kandratsenka, Daniel J. Auerbach, Alec M. Wodtke, Hua Guo, G. Barratt Park

**Affiliations:** † Institute for Physical Chemistry, University of Göttingen, Tammannstraße 6, Göttingen 37077, Germany; ‡ Department of Dynamics at Surfaces, Max Planck Institute for Multidisciplinary Sciences, Am Faßberg 11, Göttingen 37077, Germany; § Department of Chemistry and Chemical Biology, University of New Mexico, Albuquerque, New Mexico 87131, United States; ∥ International Center for Advanced Studies of Energy Conversion, University of Göttingen, Tammannstraße 6, Göttingen 37077, Germany; ⊥ Department of Chemistry and Biochemistry, Texas Tech University, Box 41061, Lubbock, Texas 79409, United States

## Abstract

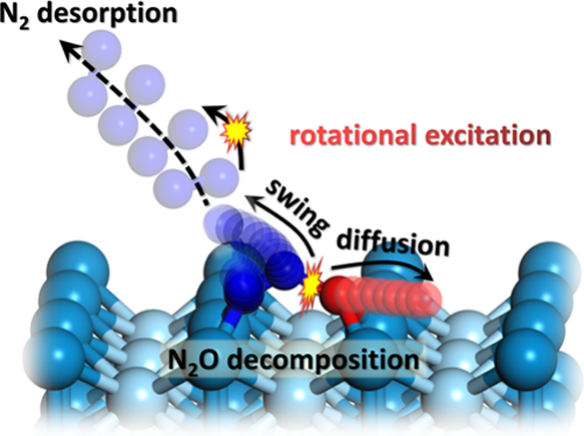

We employ time-slice and velocity map ion imaging methods to explore the quantum-state resolved dynamics in thermal N_2_O decomposition on Pd(110). We observe two reaction channels: a thermal channel that is ascribed to N_2_ products initially trapped at surface defects and a hyperthermal channel involving a direct release of N_2_ to the gas phase from N_2_O adsorbed on bridge sites oriented along the [001] azimuth. The hyperthermal N_2_ is highly rotationally excited up to *J* = 52 (*v*″ = 0) with a large average translational energy of 0.62 eV. Between 35 and 79% of the estimated barrier energy (1.5 eV) released upon dissociation of the transition state (TS) is taken up by the desorbed hyperthermal N_2_. The observed attributes of the hyperthermal channel are interpreted by post-transition-state classical trajectories on a density functional theory-based high-dimensional potential energy surface. The energy disposal pattern is rationalized by the sudden vector projection model, which attributes to unique features of the TS. Applying detailed balance, we predict that in the reverse Eley–Rideal reaction, both N_2_ translational and rotational excitation promote N_2_O formation.

## 1 Introduction

To understand the dynamics of exoergic chemical reactions at surfaces, which may produce hot products, it is essential to elucidate energy transfer to the solid. When energy transfer is efficient, the products may rapidly reach equilibrium with the surface, and the products’ energy content can be obtained from equilibrium statistical mechanics. On the other hand, if the energy dissipation is inefficient, gas phase products may be produced with large amounts of internal and translational energy. This is seen for recombinative desorption of hydrogen from Copper for example, where dynamical effects produce vibrationally and rotationally excited products^1,2^ with preferential alignment.^3^ Surface reactions involving heavier atoms may be able to exchange energy with the surface in more complex ways than do hydrogen atoms. For example, vibrationally excited CO was observed to trap to a Au(111) surface and desorb with a thermally equilibrated kinetic energy distribution without vibrational equilibration.^4^ Fundamental understanding of the mechanisms controlling the energy transfer remains elusive. Achieving a better understanding of the competition between reactivity and energy dissipation processes requires precise experimental measurements and accurate theoretical calculations, a topic that has attracted much recent attention.^5−7^

A desirable approach to this problem involves experiments, in which product translational energy, angular, and quantum-state distributions are accurately measured.^8^ Such experiments open up an opportunity for understanding the potential energy surface (PES) governing both the reaction and energy dissipation, including gaining insights into nature of the reactive transition state (TS). While methods for performing spectroscopy of the TS are established for gas phase reactions,^9^experimental methods capable of providing information about the TSs of reactions at surfaces are still quite limited. In CO oxidation on Pd(110), for example, the angle-resolved vibrational and rotational excitation of the CO_2_ product observed using infrared chemiluminescence provided valuable information about the various TS configurations and how they depend on the surface structure and adsorption patterns of the reactants.^10^ In a more recent study of CO oxidation on Pt surfaces, thermal and hyperthermal channels were identified and attributed to different TSs corresponding to reactions at terrace and step sites.^11,12^ The TS has also been investigated using ultrafast pump–probe spectroscopy.^13^ Here, the CO oxidation reaction on Ru(0001) was initiated by exciting the surface with an optical pulse, and the TS was probed via its transient X-ray spectrum. Another approach to probing the TS is to measure the internal state population distributions of the desorption products.^14,15^

This paper presents experimentally obtained angle-resolved translational energy and quantum-state population distributions for the N_2_ products of nitrous oxide reduction at Pd(110)
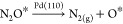


Here, asterisks indicate adsorbates. These observations are explained by an in-depth theoretical analysis of the role of the TS in N_2_O decomposition on Pd(110). Such synergistic experimental and theoretical interplay goes beyond previous work on this system^16−22^ and helps shed light on the nature of the reaction’s TS, its related decomposition dynamics, and the accompanying energy transfer.

## 2 Methods

The experiments were performed on a modified version of an instrument reported previously,^23^ and further details of both the experimental and theoretical methods used in this work are described in the Supporting Information. Briefly, N_2_O and CO are dosed onto the Pd(110) surface using two pulsed molecular beams, and reaction products are detected in the gas phase using an ion-imaging detector equipped with a time-of-flight (TOF) mass spectrometer (Figure S1). CO was used to remove adsorbed oxygen atoms exploiting the reaction CO* + O* → CO_2(g)_. This avoids poisoning of the surface from the N_2_O reduction reaction and allows us to carry out N_2_O decomposition at a controlled surface coverage of O atoms (O-coverage). We carefully controlled the O-coverage because the excess O adatoms might induce the reconstruction of Pd(110) to a 2 × 1 missing-row surface structure^24,25^ and occupy the adsorption sites of N_2_O on Pd(110), leading to a drop of the flux of hyperthermal N_2_ as the surface sites for the N_2_O dissociation become unavailable. We checked the surface structure by ex situ post coverage-control measurements using low energy electron diffraction (LEED). Under the low O-coverage conditions reported here, we did not observe the 2 × 1 LEED pattern that signifies the reconstruction of the Pd(110) surface. We also did not see carbon deposition on the surface via Auger electron spectroscopy. The combination of resonance-enhanced multiphoton ionization (REMPI) with ion imaging provides angular and velocity distributions of the N_2(g)_ product in specific quantum states. Density functional theory (DFT) calculations were carried out using VASP^26,27^ with the Perdew–Burke–Ernzerhof (PBE)^28^ functional, and a PES was constructed using a neural network approach,^29,30^ and then modified based on new experimental data to correct the commonly seen overbinding problem.^31^ The dynamics were investigated on the modified PES using classical trajectories initiated at the decomposition of the TS.^12^ Details of the DFT calculations, the fitting and modification of the PES, and the dynamical calculations are provided in the Supporting Information.

## 3 Results

**Figure 1:**
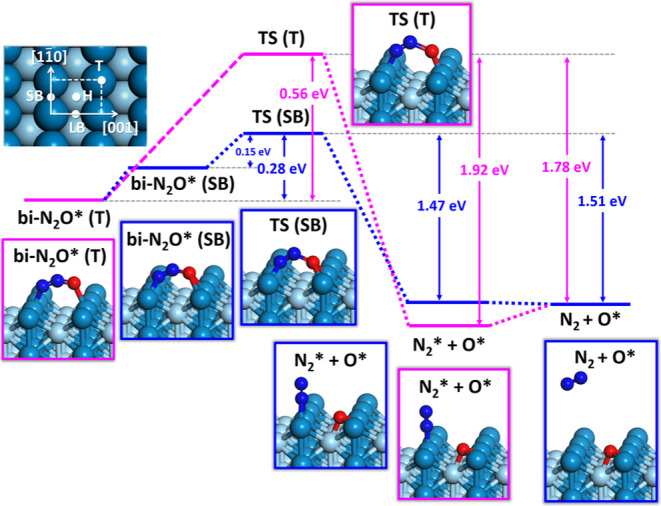
Energy diagram of N_2_O decomposition pathways on the Pd(110) surface. The decomposition of N_2_O from its most stable bidentate adsorption configuration at the top (T) site along the [001] azimuth follows the TS at the short bridge (SB) sites (blue pathway), which is significantly lower than the TS at the top sites (purple pathway). The decomposition releases 1.47 eV of energy for forming a transient physisorbed N_2_* and chemisorbed O*, followed by the desorption of N_2_. Energies cited here are from the empirically modified PES, as detailed in the Supporting Information.

DFT calculations using the PBE functional predicted an adsorption energy of 0.5 eV for N_2_ on Pd(110), which significantly overestimates the binding energy derived from temperature-programmed desorption (∼0.2 eV) (see the Supporting Information). To correct the overbinding of N_2_, we have modified the DFT PES by reducing the N_2_ adsorption energy to 0.2 eV, which simultaneously has the effect of increasing the reaction barriers by ∼0.3 eV. The increased barrier heights are reasonable as functionals containing GGA exchange are known to systematically underestimate reaction barrier heights for systems for which the work function of the surface minus the electron affinity of the molecule is less than 7 eV,^32^ a condition obeyed by N_2_O + Pd(110). This suggests that the electronic structure of the N_2_O/Pd(110) system is less well described by the PBE GGA functional used here in the region of the PES for which trajectories are initiated and for which our sudden vector projection (SVP) model analysis is performed (see below and the Supporting Information). Figure 1 shows the DFT bidentate structures of adsorbed N_2_O that may dissociate over relatively low barriers to form gas-phase N_2_ and adsorbed O atoms (note that the most stable structure of the adsorbed N_2_O is an N-bound monodentate configuration that cannot readily eject N_2_ into the gas phase). The lowest energy bidentate structure exhibits bonding at the top site [as shown in Figure 1 bi-N_2_O* (T)]. Interestingly, the barrier for direct decomposition of this species is, however, larger than an indirect decomposition involving a similar adsorbate structure at the short bridge (SB) site (bi-N_2_O* in Figure 1). Indeed, TS (T) has a small (∼1%) Boltzmann factor at the experimental temperature. This suggests that in the experiment, adsorbed N_2_O finds its way to the SB site before decomposing and releasing ∼1.47 eV of energy. This is supported by DFT calculations, showing no barrier to diffusion from bi-N_2_O* (T) to bi-N_2_O* (SB) except the energicity. The TS of the reaction [TS (SB) in Figure 1] exhibits an N–N–O angle (∠NNO) of 124.3° and an N–O bond length (*r*_NO_) of 1.507 Å, which can be compared to the geometry of the initial state of bi-N_2_O* (∠NNO = 141.6°, *r*_NO_ = 1.27 Å). All geometries are listed in Table S1.

**Figure 2:**
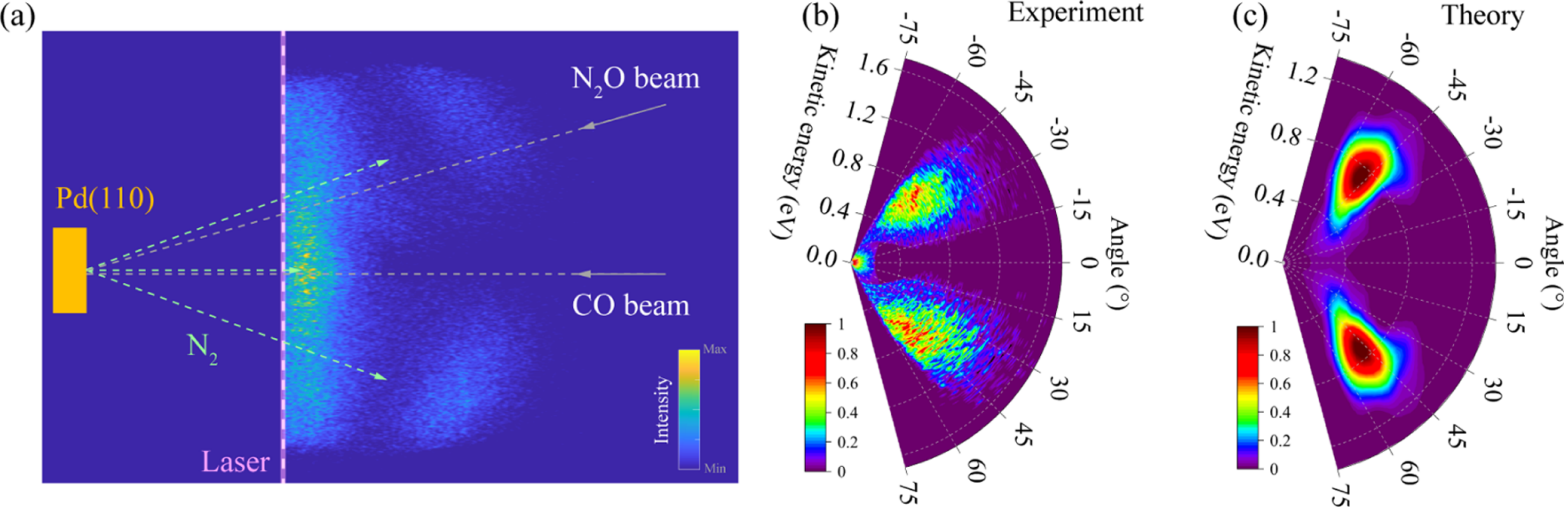
Bimodal distributions of N_2_ in a selected quantum state *J* = 10 ( *v*″ = 0), produced by N_2_O reduction on Pd(110) at *T*_surf_ = 550 K. (a) Slice ion imaging of a N_2_ product. The laser is a pulsed, tunable UV dye laser at a wavelength of 202.36 nm, and its position is indicated by a purple dashed line. The velocity vectors of incident N_2_O and CO beams are labeled by the gray arrows. The velocity vectors of the N_2_ product are shown in green arrows. The surface is shown by the yellow rectangle, in which the [001] direction is parallel to the laser beam. The O-coverage is 0.015 ML. (b) Measured angular and kinetic energy distributions of N_2_ from the ion image in panel (a) shown in polar coordinates. The angle of 0° represents the surface normal. (c) Simulated angular and kinetic energy distributions of the hyperthermal N_2_ product. The simulation is performed at 650 K and includes all *J* and *v* states.

Figure 2 shows ion imaging results detecting gas-phase N_2_ molecules produced in the surface reaction at Pd(110) when detected state selectively [N_2_ (*v*″ = 0, *J* = 10)] via (2 + 1) REMPI. Figure 2a shows the raw ion image, which was integrated over the full duration (0–120 μs) of the N_2_ product generated from a N_2_O beam pulse (see also Section S1 and Figure S2a in Supporting Information). This was accomplished by varying the delay between the pulsed N_2_O beam and the pulsed REMPI laser.^11^Figure 2b shows the angle-resolved translational energy distribution obtained from this ion image after the appropriate transformation (Supporting Information). One clearly sees two high-energy lobes—the average translation energy (*E̅*_trans_) is 0.62 eV—with angular distributions sharply peaked at +45° and −45° with respect to the surface normal. A third feature exhibits very low energies and a broad angular distribution peaked at the surface normal. Hereafter, we refer to a hyperthermal and a thermal channel. The detailed results for the thermal channel, though not the focus of this work, are discussed in Sections S2c and S3d in the Supporting Information. It is interesting that the thermal channel has not been seen in previous experiments that used a cross-correlation TOF technique. This could be the result of the geometry of those instruments,^17,33^ underscoring the value of ion imaging in the study of surface reactions. Figure 2c shows the results of post-TS dynamics calculations on a high-dimensional, modified PES based on the embedded atom neural network representation^30,37^ of DFT energies in the reaction channel (see Section S3). Specifically, classical trajectories were initiated at the TS (SB) configuration with the Boltzmann distribution at the temperature of the surface (*T*_surf_). The resulting angle-resolved translational energy distribution is quite similar to that seen in the experiment—note that the simulation is integrated over all internal energies of the N_2_ molecule. The angular distribution obtained on the unmodified PES peaks at a much larger (∼70°) angle than that seen in the experiment.

**Figure 3:**
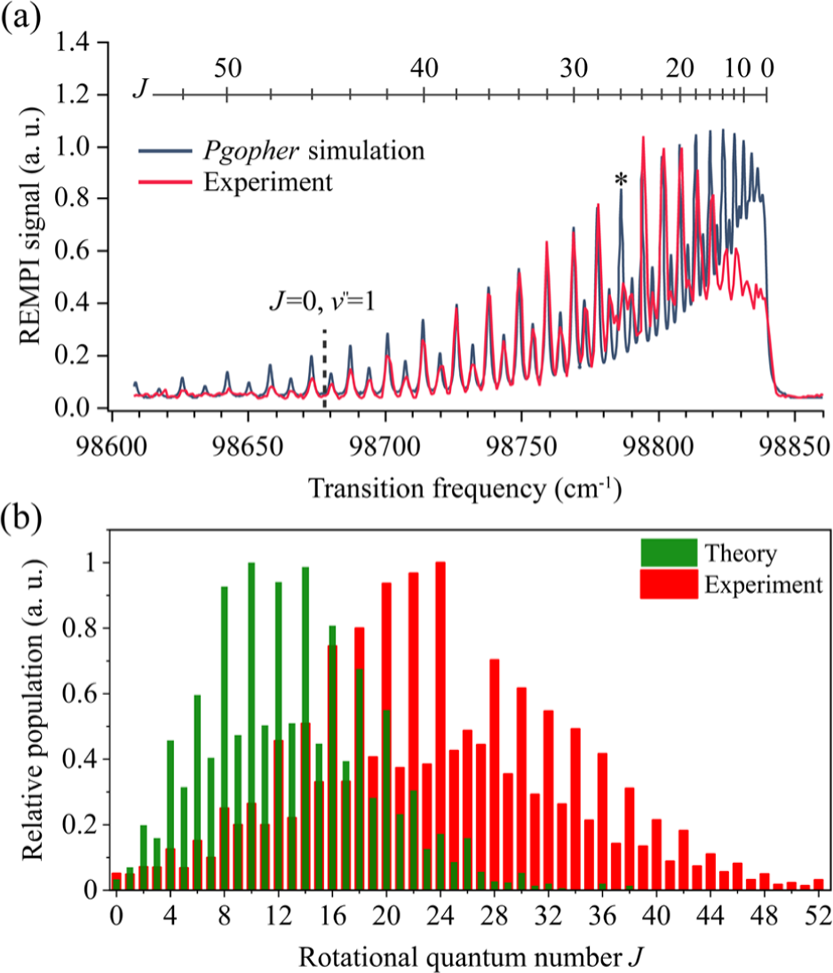
Rotational excitation of N_2_ in the hyperthermal channel of N_2_O decomposition on Pd(110). (a) (2 + 1) REMPI excitation spectrum (red curve) obtained via the two-photon-resonant *a*″^1^Σ_**g**_^+^(*v*′ = 0) ← *X*^1^Σ_**g**_^+^ (*v*″ = 0) transition. The vertical dash-line indicates the expected position for the N_2_ vibrationally excited state (*J* = 0, *v*″ = 1). The asterisk notes the *J* = 26 transition, whose intensity is influenced by a perturbation. The experimental spectrum is compared to a simulated spectrum with a thermal population distribution (*T*_rot_ = 1950 K) (black). The simulation used *PGopher*(34) with known measured spectroscopic constants.^35,36^ The combs indicate the rotational states labeled by *J*. The *x*-axis gives the wavenumber corresponding to the two-photon excitation energy. *T*_surf_ is 650 K, and the O-coverage is 0.13 ML. (b) Comparison of experimentally derived and theoretically predicted rotational population distributions.

Figure 3a shows the REMPI spectrum obtained when selectively detecting the N_2_ product with hyperthermal velocities (see Section S1 and Figure S2). All of the transitions belong to the Q-branch (Δ*J* = 0) of the two-photon *a*″^1^Σ_**g**_^+^(*v*′ = 0) ← *X*^1^Σ_**g**_^+^ (*v*″ = 0) transition. Molecules are detected with high rotational excitation (*J* > 50). We easily excluded the possibility that the high rotational excitations derive from either (i) the scattering of N_2_ (as a trace contaminant in N_2_O incoming beam), which has the rotation temperature (*T*_rot_) of 700 K comparable to the *T*_surf_ (Figure S4d) or (ii) the N_2_ produced by one-color photodissociation of N_2_O in a supersonic beam at 203–205 nm, which peaks at *J* = 74 but exhibits no population in the range *J* = 40 to 50 (*v*″ = 0).^38^ Due to the nuclear spin statistics for nitrogen molecule,^39^ we observe an even-odd intensity alternation. The *J* = 26 transition, marked with an asterisk, has an anomalous intensity due to a perturbation arising from a vibrational level in the ^1^Σ_**g**_^+^(II) outer well state of N_2_,^35,40^ the effects of which are not included in the *PGopher* simulation^34^ (black curve in Figure 3a). The desorbing N_2_ molecules exhibit a non-Boltzmann rotational state population distribution, as evidenced by the disagreement in the *J* = 0–14 region between the observed intensities and those predicted from the *PGopher* simulation obtained at the *T*_rot_ of 1950 K. The vibrational excitation of N_2_ is not observed as there is no evidence of the *a*″^1^***Σ***_**g**_^+^(*v*′ = 1) ← *X*^1^Σ_**g**_^+^ (*v*″ = 1) transition, whose position is indicated by the dashed line in Figure 3a. Since both of the Franck–Condon factors for the *v*′ = 1 ← *v*″ = 1 transition and the observed *v*′ = 0 ← *v*″ = 0 transition are the same and both close to unity,^35,40^ the REMPI spectroscopy should provide similar sensitivity to both vibrational states. Figure 3b shows a comparison of the experimental results to those obtained from classical trajectory simulations. The simulations also show substantial rotational excitation of N_2_—the average rotation excitation is 0.052 eV—that is significantly less than that seen in the experiment (0.167 eV), suggesting that the torque at the TS is underestimated or that too much rotational energy is lost during the post-TS dynamics. The experimental data analysis is described in Section S2a and Figure S3.

**Figure 4:**
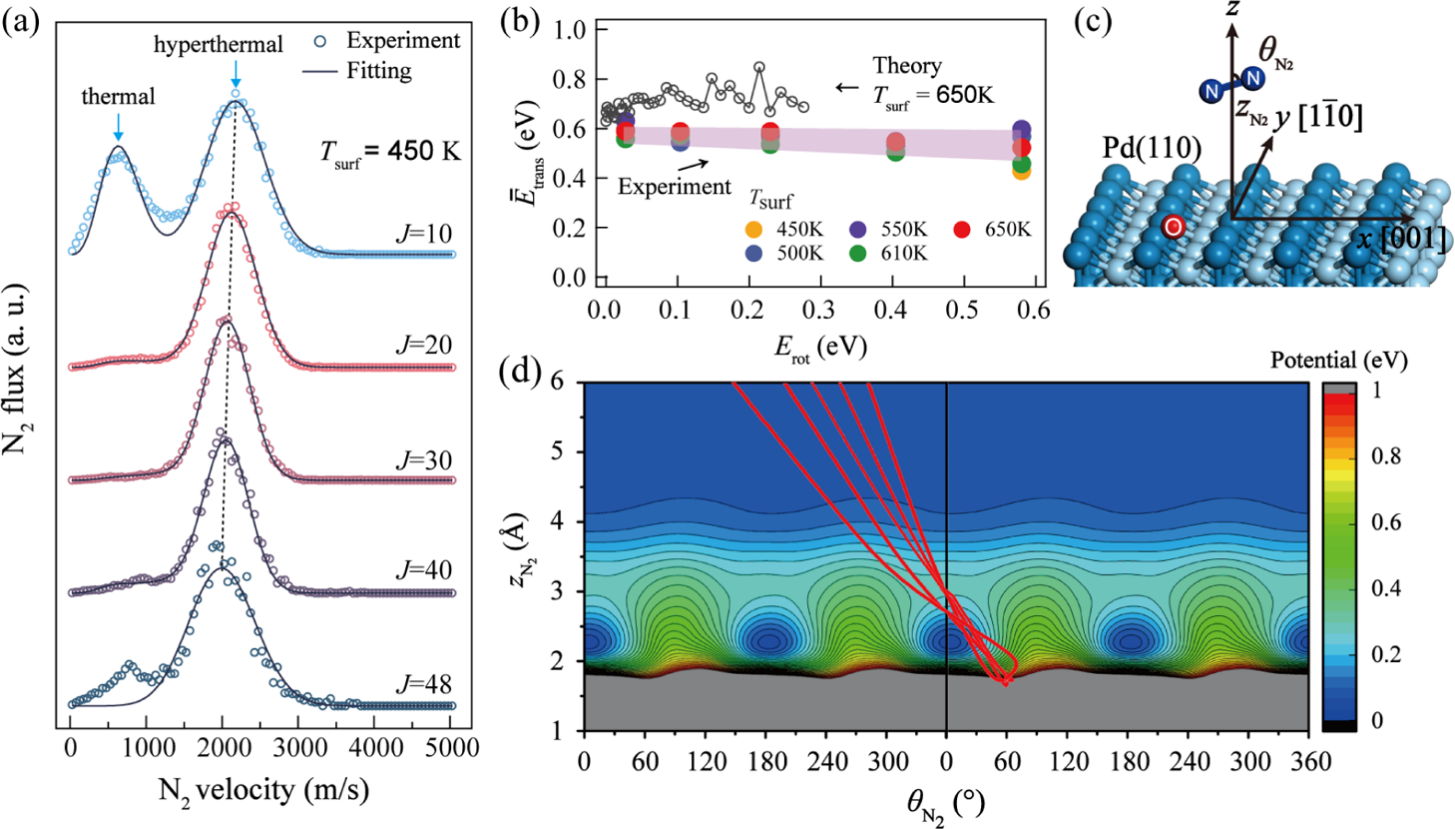
Velocity distributions and rotational energy of N_2_ obtained from velocity-resolved and state-resolved measurements for the N_2_O decomposition on Pd(110). (a) Velocity distributions of desorbing N_2_ in different rotational states of the ground vibrational state (*v*″ = 0, *J*) at *T*_surf_ = 450 K. The O-coverage is estimated to be 0.007 ML. The dashed line is to guide the eyes to highlight the slight shift in the peak position. (b) Experimental and theoretical results for the correlation of the average translational energy (*E̅*_trans_) and the rotational energy (*E*_rot_). The experimental measurements are carried out at *T*_surf_ = 450–650 K, and the theoretical calculations are performed at 650 K. (c) Definition of the Jacobi coordinates for the desorption of N_2_. (d) Typical desorption trajectories (red lines) superimposed on the PES contours reflect little coupling between the two coordinates.

Figure 4a shows velocity distributions for N_2_ appearing in five selected rotational states, revealing how the contribution of thermal and hyperthermal reactions varies with *J*. Here, the thermal channel is fitted with a Maxwell–Boltzmann (MB) function, and the hyperthermal channel is fitted with a streaming MB function; see Section S2b. In the *J* = 10 velocity distribution, the thermal N_2_ exhibits a translational temperature of 450 ± 25 K, similar to the surface temperature (450 K). The thermal channel is not seen for higher *J* states—note that the low-velocity feature in the *J* = 48 velocity distribution arises from background. The *E̅*_trans_ of the hyperthermal channel is plotted as a function of rotational excitation and for several surface temperatures, as shown by solid circles in Figure 4b. Here, the well-known rotational constants for N_2_ were used.^41^ The *E̅*_trans_ is nearly independent of surface temperature and remains nearly constant as rotational excitation increases. The trajectory simulations (open circles) reproduce the trend well. Figure 4d shows exemplary trajectories in the two Jacobi coordinates (defined in Figure 4c) superimposed upon the PES. The red straightline trajectories indicate that the translational and rotational degrees of freedom of the desorbing N_2_ are largely decoupled from one another, leading to rotational excitation that is nearly independent of translational excitation.

## 4 Discussion

In this work, we have experimentally determined the state- and angle-resolved N_2_ translational energy distributions for the hyperthermal channel of N_2_O decomposition on Pd(110). The angular and translational energy distributions of the hyperthermal channel are qualitatively similar to previous experimental observations.^16−22^ In this study, the O* and CO* species almost have no effects on the N_2_ desorption dynamics but may block the active sites for N_2_O decomposition, consistent with the results from previous reports.^17,19^ The determination of the N_2_ rotational state distribution reported in this work bridged the gap in our understanding of the reaction dynamics. The simulations shown in Figures 2c and 4b are in reasonably good agreement with the experimental observations. Both experiments and simulations in Figure 2b,c show that the kinetic distributions of the hyperthermal N_2_ mainly range from 0.4 to 1.2 eV within desorption angles between 20 and 60° with respect to the surface normal. The computed *E̅*_trans_ in Figure 4b, while somewhat larger, is insensitive to the rotational excitation as per the trend observed by the experiments. These results support the hypothesis that the hyperthermal channel originates from direct decomposition of a bidentate adsorbate bound at the SB site—see Figure 1. Our simulations from TS (T) yielded a cosine-like angular distribution, which is inconsistent with experimental observations in the hyperthermal channel.

The observed energy disposal in the decomposition of N_2_O can be understood with the SVP model,^42^ which attributes the excitation of an N_2_ mode to its coupling with the reaction coordinate at the TS. As shown in Table 1, both the translational and rotational modes of N_2_ are strongly coupled with the reaction coordinate, allowing facile energy flow into these two modes. The vibrational mode, on the other hand, has relatively weak coupling, resulting in little vibrational excitation. The SVP results can be readily understood if one considers the consequences of the N–O bond cleavage, the recoil of the N_2_ and O moieties leads to a strong torque in the rotational coordinate and repulsion in the desorption and diffusion (along [001]) coordinates. However, compared to the SVP results which suggested a strong torque at the TS, our dynamics simulations show a significant underestimation of the N_2_ rotational excitation, suggesting that additional dynamics in the exit channel must be significant. This discrepancy indicates possible inaccuracies in the PES in the exit channel.

**Table 1:** SVP Values of the N_2_ Vibrational, Rotational, and the Translational Modes onto the Reaction Coordinate^a^

mode		SVP
N_2_ vibrational mode	*v*	0.131
N_2_ rotational mode	*j*	0.309
	*t*_*x*_ [001]	0.363
N_2_ transitional mode	*t*_*y*_ [11̅0]	0.002
	*t*_*z*_	0.257

a The *t*_*x*_, *t*_*y*_, and *t*_*z*_ represent projections of translational modes along the surface azimuth of [001], [11̅0], and surface normal, respectively.

The minimum energy reaction pathway on the modified PES shows that the available energy is about 1.5 eV (Figure 1). This is substantially higher than the experimentally observed sum of the average translational and rotational energy released (∼0.8 eV). This indicates that, on average, about 47% of the energy released from the reaction barrier flows elsewhere, mainly producing excited phonons and a hot nascent O*. The nascent N_2_ is unlikely to lose energy to electron–hole pair excitation because N_2_ molecule has a closed shell electronic structure which does not easily interact with the substrate electrons and thus behaves in an electronically adiabatic manner.^43^ The eventual equilibration may involve ballistic diffusion of the O atom to a neighboring Pd row; see Section S3d. The fraction of available energy transferred to the surface (0.47) can be compared to that in reactive CO_2_ desorption due to formate decomposition on Cu surfaces^44,45^ and CO oxidation on the Pt surfaces.^11,46^ In those systems, the fraction of available energy transferred to the solid was estimated at 0.2 and 0.5, respectively.

The lack of a correlation between rotational and translational excitation also sheds light on the energy-transfer dynamics between the surface and the reactive complex. If there were no energy transfer to the surface, including the energy coupling between N_2_ and O*, during the N_2_O dissociation on the surface, we would observe a strong anti-correlation between translation and rotation—the slope of the plots in Figure 4b would be −1. This is clearly inconsistent with both our experimental and theoretical results. Figure 4b shows that the final N_2_ translational energy is independent of its final rotational energy; this implies that the amount of energy that flows into the O*/Pd(110) surface decreases as the *E*_rot_ increases.

We found that the rotational excitation of the N_2_ was not influenced by the incidence energy of N_2_O (Figure S5), indicating that the adsorbed N_2_O thermalizes with the solid prior to decomposition. The rotational excitation increases slightly—from 0.14 to 0.17 eV—with increasing *T*_surf_ from 450 to 700 K and with increasing O-coverage from 0 to 0.22 ML (Figure S6). The weak surface temperature dependence suggests that excited surface phonons do not significantly affect energy exchange between the reaction complex and the solid during the desorption of hyperthermal N_2_. The Pd(110) surface reconstruction with “missing-rows”—or partially reconstructed domains may appear when the O-coverage reaches 0.22 ML according to previous reports.^17,24,47^ Nevertheless, the rotational excitation, angular distributions, and velocity distributions of the hyperthermal N_2_ are found to be insensitive to the O-coverages and surface temperatures (Figures 4b and S6).^17^ This suggests that the local site for the N_2_O decomposition in the hyperthermal channel is the same as the unreconstructed structure of Pd(110),^48^ and the active form of N_2_O must have a bridge adsorption structure as the tilted monodentate N_2_O adsorbed on Pd(110) is not favorable.^16,25^

It is also interesting to compare the observations of this work with other surface reactions forming gas phase N_2_, which have different TS characters. Although this reaction produces highly rotationally hot but vibrationally cold N_2_, the recombination of two N-atoms (N* + N*→ N_2(g)_) on Cu(111)^49^ produced both vibrational and rotational excitation. This is presumably because the N_2_ molecule experiences a relatively long-range torque from the TS with an extended N–N bond. In another example, NH_3_ cracking on Ru(001) produces vibrationally hot but rotationally cold N_2(g)_, also suggesting that the bond length of N–N plays an important role in the recombinative desorption.^50^ For bimolecular reactions, the N_2(g)_ product from NO + NH_3_ on Pt(100) is vibrationally and rotationally excited, whereas the N_2(g)_ from NO + H_2_ on the same surface is rotationally equilibrated with the surface and has no vibrational excitation,^51^ indicating that both of the TS structures and reaction mechanisms are different for the N_2(g)_ formation in these two reactions. In contrast, the N_2(g)_ from NO + H_2_ on Pd(110) is vibrationally excited but does not possess any excess translational or rotational excitation^52^ because the nascent N_2_ remains transiently trapped at the surface but desorbs before complete accommodation of N–N stretching excitation. The product energy partitioning observed in the current work is markedly different.

## 5 Conclusions

In this study, ion imaging methods were combined with theoretical calculations to elucidate the surface reaction dynamics of N_2_O decomposition on Pd(110). In the hyperthermal channel, our results not only confirmed previous observations that the N_2_ exhibits anisotropic and sharp angular distributions with high translational energies but also revealed that the N_2_ product is highly rotationally excited up to *J* = 52 and is exclusively produced in *v*″ = 0. Furthermore, the rotational excitation is independent of the translational excitation and surface temperature. On average, about 50% of the barrier energy released at the TS (1.5 eV) is deposited into the desorbing N_2_, and half remains at the surface. These experimental observations were interpreted based on trajectory simulations of the post-TS dynamics on a machine-learned PES. The rotational excitation and hyperthermal kinetic energy distributions of the N_2_ product are shown to originate from decomposition via the bidentate TS (SB). On the basis of detailed balance, these state-resolved results suggest that both rotational and translational excitation of N_2_ is needed to promote the formation of adsorbed N_2_O on an O-covered Pd surface. It is worth noting that bimodality in such chemical reactions often represents multiple competing kinetic pathways. We also found a thermal channel not previously seen; we speculate that this may result from N_2_ that can be trapped at the surface when formed at defect sites.

## Supporting Information Available

Experimental methods, data analysis methods, and theoretical methods (PDF)
