# Supplementary material for: Highly Rotationally Excited N2 Reveals Transition-State Character in the Thermal Decomposition of N2O on Pd(110)
Source: J Am Chem Soc. 2023 May 24;145(22):12044–50. doi: 10.1021/jacs.3c01127 (PMC10251513; doi:10.1021/jacs.3c01127)
Supplement: Supplementary file 1 — ja3c01127_si_001.pdf [file ja3c01127_si_001.pdf]

Supporting Information

for

Highly Rotationally Excited N_2 Reveals Transition State Character in the Thermal Decomposition of N_2O on Pd(110)

Jiamei Quan[†,‡,#], Rongrong Yin[§,#], Zibo Zhao[†,‡], Ximei Yang[†,‡], Alexander Kandratsenka[‡], Daniel J. Auerbach[‡], Alec M. Wodtke[†,‡,‖], Hua Guo[§,*] and G. Barratt Park[‡,⊥,*]

† Institute for Physical Chemistry, University of Göttingen, Tammannstraße 6, 37077 Göttingen, Germany

‡ Department of Dynamics at Surfaces, Max Planck Institute for Multidisciplinary Sciences, Am Faßberg 11, 37077 Göttingen, Germany

§ Department of Chemistry and Chemical Biology, University of New Mexico, Albuquerque, New Mexico 87131, USA

‖ International Center for Advanced Studies of Energy Conversion, University of Göttingen, Tammannstraße 6, 37077 Göttingen, Germany

⊥ Department of Chemistry and Biochemistry, Texas Tech University, Box 41061, Lubbock, TX 79409, USA

# these authors contributed equally.

* corresponding authors: hguo@unm.edu; barratt.park@ttu.edu

S1. Experimental Methods

We used a modified version of the "mobile beamer",[1] replacing the atomic beam source with a dual molecular beam source for dosing N_2O and CO on the surface, followed by detection via time-of-flight (TOF) slice ion or velocity map imaging. See Fig. S1. This apparatus is composed of two source chambers (a main and a side beam), two differential chambers (I and II) and a reaction chamber (base pressure $\gtrsim 2 \times 10^{-10}$ mbar). In the present study, a palladium crystal (MaTeck GmbH) had a diameter of 10 mm. The uncertainty of cut-angle for the Pd(110) crystal surface is specified to be < 0.1%. The sample was pretreated by cycles of Ar^+ sputtering for 15 minutes at room temperature and annealing for 20 minutes at 710 K. The surface crystallinity and cleanliness were confirmed by low energy electron diffraction (LEED) and Auger electron spectroscopy (AES) in the reaction chamber. The incident CO beam composed of 1% CO (purity 99.99%) in Helium (purity 99.997%) was introduced along the surface normal. The N_2O beam was prepared with 5% N_2O (purity 99.95%) diluted in the Helium, incident at 30° to the surface normal. For the N_2O beam, we used a home-made pulsed nozzle based on the Even-Lavie design[2] operated with 8 bar backing pressure. The molecular expansion entered the differential chamber I after passing through a skimmer and differential chamber II after passing through an aperture. The pulsed molecular CO beam was prepared separately in the same way. The two pulsed beams crossed at a spot with a diameter of about 7 mm on the center of the palladium surface.

For steady-state measurements, the N_2O beam fired at a constant repetition rate (e.g., 250 Hz) to generate the N_2 and the oxygen adatoms. The CO beam's repetition rate was varied between 5 and 200 Hz to control the coverage of oxygen adatoms (O-coverage) at the surface. The O-coverage was measured by a titration method described previously.[3-4] The N_2O pulse initiated the production of N_2 and was consumed before the next N_2O pulse arrives. Combining the synchronized pulsed-laser resonance-enhanced multiphoton ionization (REMPI) with the molecular beams and the ion detector system, REMPI spectra of N_2 product were detected.

The laser generated ions were extracted perpendicular to the imaging plane by a grounded extractor grid (a diameter of 45 mm) and a repeller electrode. The imaging plane was defined by the laser beam propagation direction and the incident molecular beams (**Fig. S1**), which allows both the speed and the angular distribution to be recorded simultaneously in the ion image as shown in **Fig. 2a**. The ions flew to a z-stack set of three microchannel plates (MCPs) through a TOF tube with a length of 44 cm. An open ring electrode (Einzel lens) was equipped in the TOF tube between the extractor grid and detector and used for velocity map imaging. With delayed extraction, we achieved "slice imaging" on a phosphor screen placed behind the MCPs. The imaging was recorded by a CCD camera that viewed the phosphor screen. The MCPs experienced a voltage-pulse at a controlled delay with respect to the delayed extraction to ensure selective detection of m/z=28 (N_2^+). Two-dimensional velocity and angular distributions were recorded in a spatial imaging mode when the Einzel lens was grounded. Velocity map imaging was possible when the appropriate voltage was applied to the Einzel lens. The ion intensity shown in the raw ion images was proportional to the density of molecules in the ionization volume. Density-to-flux transformation was accomplished by use of the measured velocities.

For the (2+1) REMPI experiments, light from 202.33 to 202.82 nm was obtained from a tunable dye laser with third harmonic generation capability (Sirah Cobra Stretch CBST-G-18+THU-205-N). The laser was operated with Rhodamine B, and Rhodamine 101 dyes and pumped by the second harmonic of a Nd:YAG laser (Innolas Spitlight 600 S/N P1754) operated at a repetition rate of 50 Hz. The fundamental laser pulse was frequency tripled by a pair of BBO crystals producing 8 ns pulses with 0.1−0.6 mJ/pulse and a linewidth of 0.5 cm^{-1}. Wavelength calibration of the dye laser was performed with a wavemeter (HighFinesse-Angstrom WS/7). The laser beam was focused by a 250 mm lens into the region between the repeller and extractor of the detector. The focusing lens was mounted on a motorized translation stage so that the focus could be scanned along the laser propagation direction.

The velocity-resolved signals of N_2 product were recorded by varying the time delay between the ionizing laser and the opening time of the pulsed nozzle for the N_2O molecular beam. Since we know the velocity of N_2O beam and the distance between the nozzle and the surface, the arrival time of N_2O at the surface can be obtained. Similarly, since we obtained the velocity of all detected N_2 molecules because we know the distance between the surface and the detector, we can compute the time that the N_2 product left the surface. This allows a determination of the reaction time at the surface. **Fig. S2a** shows the integrated ion signal of hyperthermal N_2 covering a narrow velocity range of 1200–2700 m/s

as a function of the reaction time. This profile (open circles) is nearly identical to the shape of the incoming N_2O pulsed molecular beam, indicating that the temporal resolution of the apparatus is not good enough to observe the full kinetics of the N_2O decomposition reaction. The (2+1) REMPI spectra of hyperthermal N_2 shown in **Fig. S2b−c** were recorded in velocity-mapping mode at the delays of 38.6, 53.6, and 68.6 μs, respectively, as indicated by the vertical lines in **Fig. S2a**. The relative population distributions of three REMPI spectra are comparable as they have a very similar average rotational energy ($\bar{E}_{\mathrm{rot}}$ =0.167 ± 0.003 eV), indicating that hyperthermal N_2 produced at different reaction times has the same rotational energy distribution. The REMPI spectra were corrected for the measured fluctuations in laser energy.

S2. Data Analysis Methods

a. Average Rotational Excitation Obtained from REMPI Spectra

Using the data of **Fig. 3a,** we show how to determine the average rotational energy of hyperthermal N_2. The relative population of each J state, as shown in **Fig. S3,** are taken from the normalized peak area of a Gaussian that has been fitted to each J-specific transition. The Gaussians have a FWHM of 2.71 ± 0.024 cm^{-1}. The spectrum was normalized to the peak intensity of the $J = 22$ feature (**Fig. 3a**). The average rotational excitation $\bar{E}_{\mathrm{rot}}$ was calculated by

$$\bar{E}_{\mathrm{rot}} = \sum_{i=1}^{i=n}((B_0 J_i(J_i+1) - D_0 J_i^2 (J_i+1)^2)\cdot N_i) \,/\, \sum_{i=1}^{i=n} N_i, \tag{S1}$$

Here, the rotational constant B_0 is 1.9897 ± 0.0003 cm^{-1} and centrifugal distortion constant D_0 is (6.1 ± 0.5) × 10^{-6} cm^{-1}.[5] N_i is the relative population for each J_i rotational state. We assumed the error of the relative population from the Gaussian fittings as $\Delta N_i = 10\% N_i$, the standard deviation of the average rotational energy $\Delta\bar{E}_{\mathrm{rot}}$ is thus estimated by

$$\Delta\bar{E}_{\mathrm{rot}} = \sqrt{\sum_{i=1}^{i=n}(B_0 J_i(J_i+1) - D_0 J_i^2 (J_i+1)^2)^2 \cdot \left(\frac{1}{\sum_{i=1}^{i=n} N_i} - \frac{N_i}{(\sum_{i=1}^{i=n} N_i)^2}\right)^2 \cdot \Delta N_i^2}\ . \tag{S2}$$

The unit of $\bar{E}_{\mathrm{rot}}$ in Eq. (S1) and $\Delta\bar{E}_{\mathrm{rot}}$ in Eq. (S2) are cm^{-1}.

b. N_2 Velocity Distributions

The desorbing N_2 flux distribution is bimodal as shown in **Fig. 2a**. The slow component was fit by a Maxwell-Boltzmann (MB) distribution.

$$f_{\mathrm{thm}}(v) = Av^3\exp(-mv^2/(2k_B T_0)), \tag{S3}$$

where A is the normalization factor, m is the mass of N_2 molecule, k_B is the Boltzmann constant, and T_0 is the temperature of the velocity distribution. The hyperthermal component was fit by a streaming MB distribution.

$$f_{\mathrm{hpt}}(v) = Bv^3\exp(-m(v - v_0)^2/(2k_B T_b)), \tag{S4}$$

where B is the normalization factor, v_0 is the streaming velocity and T_b is the effective temperature of the distribution. From the fits shown in **Fig. 4a**, we obtained the fitting parameters: v_0 is from 1981 m/s to 1916 m/s and T_b is ranging from 478 to 316 K for J=10 to J=40. For J=48, v_0=1654 m/s and T_b is 756 K, where fitting error exists due to the weak ion signal at J=48.

c. Thermal Component of N_2 Product

The thermal N_2 in **Fig. 2a** and **b** has a translational temperature (T_{trans}) of 450 ± 25 K, which is slightly lower than T_{surf} of 550 K. This result is consistent with desorption of N_2 from a trapped, physisorbed state—a slightly subthermal T_{trans} can be explained by detailed balance if trapping of gas-phase N_2 into the physisorption well is more probable at low incidence kinetic energies. Theoretical evidence for such a trapped state for the nascent N_2 on a defected Pd(110) surface is presented in **Fig. S13**. The REMPI spectrum of the thermal N_2 component is shown in **Fig. S4a**. The rotational temperature (T_{rot}) is about 300 K, which is much lower than T_{surf}=650 K but comparable to that of the gaseous N_2 leaking in chamber at room temperature (**Fig. S4b**).

d. Effects of C, CO, O Species and Surface Temperature

Concerning the possible effects of C, CO or O species on the surface, we have examined them carefully during the experiments. First, we did not see C deposition on the surface via Auger electron spectroscopy. Second, the results were obtained under conditions where the CO beam was asynchronous with the N_2O beam. Due to the relatively fast rates of CO desorption and oxidation, there was no CO present on the surface during the N_2O decomposition reaction. Additionally, the angular distribution of hyperthermal N_2 measured in this study is comparable with previous reported results obtained from N_2O + CO and the decomposition of adsorbed N_2O on Pd(110).[6-7] That is, the angular distributions of hyperthermal N_2 are independent of the coverages of CO or O. Finally, the $\bar{E}_{\text{rot}}$ of hyperthermal N_2 is also insensitive to the O-coverages (Fig. S6).

$\bar{E}_{\text{rot}}$ of hyperthermal N_2 increases slightly with increasing O-coverage and surface temperature (T_{surf}). As shown in **Fig. S6**, $\bar{E}_{\text{rot}}$ increases from 0.14 to 0.17 eV as T_{surf} is increased from 450 to 700 K. At each T_{surf}, $\bar{E}_{\text{rot}}$ increases by about 0.01 eV when the O-coverage is increased from 0 to 0.22 ML. In particular, $\bar{E}_{\text{rot}}$ is almost insensitive to the O-coverages below 0.13 ML.

The flux of hyperthermal N_2 decreases with increasing O-coverage at the T_{surf} of 650 K (**Fig. S7**). At high O-coverage, the bonding sites for the adsorption of N_2O available to the generation of hyperthermal N_2 are fewer than those at low oxygen coverage. This is because the excess O adatoms might induce the reconstruction of Pd(110) to a 2×1 missing-row structure and occupy the adsorption sites of N_2O (i.e. bi-N_2O* in Fig. 1) on Pd(110), leading to a drop of the flux of the hyperthermal N_2 as the surface sites for the reaction become unavailable.

Figure S8 shows the N_2 velocity distributions from the N_2O decomposition on Pd(110) with different O-coverages ranging from 0.007 to 0.216 ML at the T_{surf} of 650 K. Under our experimental conditions of low O-coverage, we mainly observed two distributions: a slow component and a fast component, which are consistent with Fig. 2a. The fast component is the hyperthermal N_2 with high rotational excitation in the ground vibrational state (v''=0).

e. Temperature-Programmed Desorption of N_2 on Pd(110)

To estimate the adsorption energy of N_2 on the Pd(110) surface, thermal desorption of N_2 was investigated with temperature-programmed desorption (TPD). As shown in **Fig. S9**, the TPD for N_2 was measured with different exposure of N_2 gas at the T_{surf} of 34 K. In the TPD profiles, four TPD peaks could be observed at *m/z*=28, which are labelled by α, β, γ and δ from the low to high desorption temperature. Note that the positions of the peaks are independent of the N_2 exposure. Therefore, we estimated the adsorption energy assuming 1st-order desorption kinetics and employing the Redhead analysis (peak maximum method)

$$E_{\text{ad}} = RT_{\text{max}}[\ln \frac{A_{\text{pre}} \times T_{\text{max}}}{r} - 3.46] \qquad \text{(S5)}$$

where the gas constant R=8.314 J/(K mol). T_{max} denotes the temperature at which the maximum desorption rate is observed and r is the heating rate (1.5 K/s). A_{pre} is the pre-exponential factor, which was assumed to lie in the range 10^{12} to 10^{14} s^{-1}. The error in the determination of the adsorption energy is obtained from the uncertainty of T_{max} which we estimate at ± 1 to ± 3 K and by considering an order of magnitude variation in the A_{pre}. The estimated adsorption energies are listed in the table of **Fig. S9** for the α, β, γ and δ peak, respectively. The small desorption activation energies of ~0.1 eV (α-state) and ~0.13 eV (β-state) suggest physisorbed states. In contrast, the desorption activation energy of 0.2 eV (γ-state) is modest; furthermore, the γ peak is narrow and symmetric which indicates a well-defined adsorption site of N_2 adsorption. As the exposure of N_2 increases, the γ peak grows accordingly. The δ peak is observed first at low exposure, reflecting an adsorbate with the strongest binding energy (~0.3 eV). This peak is relatively broad and asymmetric, representing a variety of similar adsorption sites, such as those arising from defects.

S3. Theoretical Methods

a. Density Functional Theory and Data Sampling

All periodic density functional theory (DFT) calculations were performed with the Vienna Ab initio Simulation Package (VASP).[8-9] The Perdew-Burke-Ernzerhof (PBE) functional[10] within the generalized gradient approximation (GGA) was used to describe the exchange-correlation interaction. The electron-

ion interactions were represented by the projector augmented wave (PAW) method.[11] All calculations were performed with spin polarization consideration.

The Pd(110) surface was modeled by a five-atomic-layer slab, which has a (3×3) unit cell with the top three layers relaxed, separated by a 20 Å vacuum space to avoid the inter-slab interaction. A total of 45 surface atoms were included in the unit cell, while atoms in the two bottom layers were fixed. The Brillouin zone integration was performed on a 3×4×1 Monkhorst-Pack k-point mesh. The dipole correlation in the z direction was imposed to avoid the interaction between the vertically repeated images. The wave function of the valence electrons was expanded using plane waves with an energy cutoff of 400 eV. Fermi smearing with a width parameter of 0.1 eV was used. The geometries were optimized using a conjugate-gradient method until the forces acting on each atom were less than 0.02 eV/Å. The saddle points were determined using the climbing image nudged elastic band (CI-NEB) method[12] and the dimer method,[13] and confirmed by frequency calculations.

The adsorption energies were computed according to the following equation: $E_{\mathrm{ads}} = E_{\mathrm{adsorbate/slab}} - (E_{\mathrm{adsorbate}} + E_{\mathrm{slab}})$, where $E_{\mathrm{adsorbate/slab}}$, $E_{\mathrm{adsorbate}}$, E_{slab}, are the energies of the adsorbed system, the gas-phase molecule, and the bare surface, respectively. The N_2O + Pd(110) coordinate system used to describe the N_2O decomposition process is schematically illustrated in **Figure S10**, in which the molecule configuration is defined by the N−O bond length (r_{NO}), N−N bond length (r_{NN}), the bond angle between the N−N and N−O bonds (∠NNO), the distance of the molecular center of mass (COM) to surface (z), The rotations of N_2O on the Pd(110) surface are described by the well-known three Euler angles (α, β, γ), between the Cartesian and body-fixed coordinate systems. The x and y coordinates are defined along the [001] and [$1\bar{1}0$] directions of the Pd(110) surface with the origin set at a top site in a Pd row. A total of 30 mobile atoms (27 Pd, 2 N and 1 O) were included in our model, which leads to 90 degrees of freedom.

To construct the high-dimensional PES for the N_2O + Pd(110) system, ab initio molecular dynamics (AIMD) simulations with the same DFT protocol were first performed to sample the configuration space. Following our recent work,[14] the post-transition state dynamics were described by trajectories starting from the transition state geometry. All movable atoms were assigned with the random initial velocities according to the Boltzmann distribution at an experimental temperature of 650 K. The dynamics was assumed to be in a microcanonical NVE ensemble, with constant number (N), volume (V), and energy (E). Approximately 50 trajectories were propagated each for the N_2O decomposition transition state on top and short bridge sites (TS2 in **Figure S11**) using the leapfrog algorithm in VASP. The time step was set to 1.0 fs and the trajectories were propagated until the N_2 center of mass reached 6.5 Å above the surface with the velocity pointing away from the surface or the propagation time exceeds 1 ps.

We first selected 5027 geometries out of ~60000 points from AIMD trajectories, according to their generalized Euclidian distances (GED) in terms of inter-nuclear distances and atomic forces.[15] These points were fitted to a preliminary PES, using a machine learning method described below. The PES enabled post-transition state classical trajectory simulations for different transition states on top and short bridge sites. New geometries from these trajectories were selected for DFT calculations and included in the data set based on the same GED criterion so that they were not too close to existing points. The augmented data set was then used to update the PES, followed by a new iteration of trajectory calculations, until no more points can be added.

b. Neural Network Potential Energy Surface

The AIMD approach is a powerful tool to explore the reaction dynamics. However, it is computationally expensive due to the repeated DFT calculations along the trajectories, especially for long time dynamics and rare events. A more efficient way to characterize the dynamics is to replace the DFT calculations using a machine learned high-dimensional PES.[16-17] Such PESs are necessarily high-dimensional because a sufficient number of surface atoms have to be included to account for the energy transfer accompanying the collision process.[18] The PES for the N_2O + Pd(110) system was constructed by means of the Embedded Atom Neural Network (EANN) approach.[19] In the EANN framework, the total energy of the system is regarded as the sum of atomic energies, each of which is an output of an atomic NN determined by the electron density of this atom embedded in the environment consisting of other atoms nearby,[19]

$$E = \sum_{i=1}^{N} E_i = \sum_{i=1}^{N} NN_i(\rho^i). \quad \text{(S6)}$$

For simplicity, the embedded electron density like structural descriptors (ρ^i) can be represented by Gaussian-type orbitals (GTOs) centered at neighboring atoms, resulting in multiple orbital-dependent density components,

$$\rho^i_{L,\alpha,r_s} = \sum_{l_x,l_y,l_z}^{l_x+l_y+l_z=L} \frac{L!}{l_x!l_y!l_z!} \left(\sum_{j=1}^{n_{atom}} c_j \varphi^{\alpha,r_s}_{l_x l_y l_z}(r_{ij}) f_c(r_{ij}) \right)^2, \quad \text{(S7)}$$

where n_{atom} is the total number of atoms lying nearby the embedded atom within a cutoff radius (r_c) and $f_c\,(r_{ij})$ a cutoff function[20] to ensure that the contribution of each neighbor atom decays smoothly to zero at r_c. The GTO is written as,

$$\varphi^{\alpha,r_s}_{l_x l_y l_z}(r_{ij}) = x^{l_x} y^{l_y} z^{l_z}\, exp\left(-\alpha \left| r_{ij} - r_s \right|^2\right), \quad \text{(S8)}$$

where $r_{ij} = (x, y, z)$ represents the Cartesian coordinates of the embedded atom i with atom j being the origin, r_{ij} is the distance of atom between i and j, l_x, l_y and l_z represent the angular momentum components in each axis, and their sum is the total orbital angular momentum (L), α and r_s are parameters that determine radial distributions of GTOs. Note that c_j in Eq. (S7) serves like an element-dependent expansion coefficient of an atomic orbital for atom j, which is optimized together with the element-dependent NN parameters. The EANN PES is invariant with respect to translation, rotation, and permutation.[19] The key advantage of this EANN method is that the density-like descriptors given in Eq. (S7) scale linearly with respect to the number of neighboring atoms.[21]

The final N_2O + Pd(110) PES was fitted to 12465 points with both energies and forces. These data points were divided into training and test sets with the ratio of 90:10. The hyperparameters of GTOs were L=0, 1, 2, r_c=6.0 Å, α=0.6 Å^{-2}, and Δr_s=0.58 Å, resulting in 33 structural descriptors. Each atomic NN consists of two hidden layers with 50 and 60 neurons in each. The RMSEs of the total energies and atomic forces for training and validation sets were found to be 17.57/18.15 meV, and 26.68/30.79 meV/Å, respectively. Moreover, to illustrate the fitting quality, we compared the energies and geometries of stationary points along the minimum energy path (MEP) of N_2O decomposition process with N_2O on top (T) and short bridge (SB) sites along [001] direction on Pd(110) surface optimized with DFT and EANN PES in **Figure S11**. The barrier height, adsorption well depth, and reaction energy are all well reproduced in EANN PES within 22 meV.

From **Figure S11**, we can find that the adsorption energy of N_2 on the Pd(110) surface is ~0.5 eV with the PBE functional. As described in Section S2e and **Fig. S9,** the TPD measurements yielded the adsorption energy of ~0.2 eV for N_2 adsorption (γ-state), suggesting the γ-N_2 adsorbed on Pd(110). Obviously, the adsorption energy of N_2 on Pd(110) was overestimated by the PBE functional. In order to reproduce the experimentally observed adsorption energy (~0.2 eV), the following empirical correcting potential (in eV) is added in the original EANN PES.

$$E_{\text{adj}}(Z_{\text{N}_2}) = 1.0 \times 0.00136425 \times 0.5 \times (1.0 + \tanh(1.75 \times (Z_{\text{N}_2} - 3.5))) \quad \text{(S9)}$$

As shown in **Figure S11**, the corrected PES has an N_2 adsorption energy of 0.20 eV, consistent with the TPD data. The impact of the adjustment on the overall reaction pathways is shown in the same figure.

c. Classical Trajectory Calculations

Trajectory calculations were performed with the VENUS code[22] which is heavily modified for surface processes.[23] As in the AIMD calculations, all movable atoms were assigned with the random initial velocities according to a Boltzmann distribution at an experimental temperature of 650 K. Over 2000 trajectories were propagated for each transition state using the velocity Verlet algorithm implemented in QCT. For the desorbed N_2 molecule, the vibrational quantum number was determined by Einstein−Brillouin−Keller (EBK) semi-classical quantization v,[24] and rotational quantum number J by the quantum mechanical expression for rotational angular momentum $|\vec{J}| = \sqrt{J(J+1)}\hbar$, where the $\vec{J}$ is rotational angular momentum. the translational energy (E_{trans}) of the N_2 molecule was calculated as: $E_{\text{trans}} = \frac{1}{2} m_{\text{N}_2} v^2_{\text{COM}}$, where the m_{N_2} is the N_2 mass and the v_{COM} is its center-of-mass velocity. The vibrational energy (E_v) of the N_2 molecule was determined as the difference between its internal energy

(E_{int}) and rotational energy (E_{rot}), $E_v = E_{int} - E_{rot}$. The rotational energy is given by $E_{rot} = \frac{1}{2}\vec{w} \cdot \vec{J}$, where the $\vec{w}$ is the angular velocity of N_2, the $\vec{J}$ is the rotational angular momentum of N_2. The internal energy consists of the kinetic and potential energies of N_2: $E_{int} = T + V$.

d. Additional Theoretical Results

For the N_2O molecule in the gas phase, our DFT calculations predicted a linear geometry with r_{NN}=1.146 Å and r_{NO}=1.200 Å. The most stable adsorption configuration of N_2O on Pd(110) involves monodentate linear N_2O^* (mono-N_2O^*) species, which are bonded via the terminal nitrogen with the Pd surface at the T and SB sites with tilted angles, as shown in **Figure S11**. The adsorption energy of mono-N_2O^* at T and SB is −0.323 and −0.215 eV, respectively. However, the monodentate species cannot directly decompose to N_2 and it needs to convert to a bidentate form, with the terminal N and O atoms bonding to two adjacent Pd rows on the surface. The corresponding barriers (TS1) between the two adsorption configurations, which feature a slightly bent NNO nearly parallel to the surface as shown in **Figure S11**, are quite low. At the experimental temperature, it is expected that the monodentate and bidentate species would coexist. As described in the main text, the bidentate N_2O^* species (bi-N_2O^*) are oriented along the [001] direction across two Pd rows, serving as the precursor for the decomposition. Their adsorption energies are −0.302 and −0.179 eV at the T and SB sites, respectively. These results are consistent with previous DFT studies of this system.[25] We attempted to determine the transition state between the T and SB adsorption sites, but the NEB calculations found no saddle point in between.

In order to demonstrate the quality of the EANN PES, we compared the DFT minimum energy paths (MEPs) for N_2O decomposition at the SB and T sites of the Pd (110) surface with those on the EANN PES. The DFT barriers, adsorption well depths, and reaction energies are reproduced in EANN PES within 22 meV, as shown in **Figure S11**. The EANN PES also reproduces geometries of the stationary points quite well, especially for the dissociation transition state TS2 (with a maximum deviation of 0.003 Å), as shown in **Table S1**.

Starting from the more stable bi-N_2O^* at the T site, the N_2O decomposes to N_2 and O by cleavage of the N-O bond via TS2. As shown in **Figure 1**, the barrier height for bi-N_2O^* decomposition at the SB site (0.151 eV) is significantly lower than that at the T site (0.555 eV). At the experimental temperature of 650 K, the latter is insignificant because of its small (1%) Boltzmann factor. In addition, post-transition state trajectories from TS2 at the top site yielded an angular distribution that is centered at the surface normal, due apparently to temporary trapping of the incipient N_2, which is inconsistent with the experimentally determined angular distribution for the hyperthermal channel. As a result, the preferred decomposition pathway involves the initial migration of the bi-N_2O^* from the T site to the SB site, followed by the corresponding TS2, which is shown in **Figure 1** as TS (SB). Finally, N_2 can adsorb on the Pd(110) surface in two configurations, as shown in **Figure S11**, and the conversion between the two is via TS3.

To illustrate the decomposition dynamics, the evolution of geometries, kinetic energy and rotational angular momentum (J) as a function of time for a representative trajectory from the transition state is shown in **Figure S12**. For clarity, the initial kinetic energy was set to 0 eV and surface temperature to 0 K to highlight the post-transition state dynamics. In the left panels, it is clearly seen that N−O bond rupture results in significant perturbation of the underlying Pd atoms on the surface, evidenced by large oscillatory motions of these surface atoms, due to the collision induced energy transfer from the recoiling N_2 and O*. The latter also undergoes significant diffusion motion along the surface. On the right panels, the oscillation of r_{NN} indicates the vibrational excitation in N_2 is very slight during N_2O^* decomposition process. On the other hand, the translational and rotational energies of N_2 first increase rapidly because of the highly repulsive forces induced by the N−O bond rupture. This is followed by the reduction of the translational and rotational energies as the N_2 molecule diffuses to the groove between two Pd rows, evidenced by decreasing kinetic energies. After passing through the surface groove, the translational and rotational energies of N_2 would regain because of the repulsion from the adjacent Pd row. Note that the desorbed N_2 has significant translational energy in both the perpendicular (z) and parallel (xy) components to the surface, consistent with the observed sharply off-normal angular distribution.

To explore the origin of the thermal channel, we also investigated the N_2O decomposition process on a Pd (110) defect site, modeled by a Pd (110) surface with two Pd rows missing, as shown in **Figure**

S13. The adsorption energy of N_2 at the defective Pd (110) site is calculated to be 0.65 eV, which is higher than the adsorption energy of N_2 on defect-free Pd (110), namely 0.5 eV. Based on the same adjustment of the PES, the adsorption energy would be close to 0.25 eV, which is larger than the 0.20 eV value on defect-free Pd(100). It is thus conceivable that the nascent N_2 could diffuse to a defect site and be trapped on Pd surface, leading eventually to its thermal desorption.

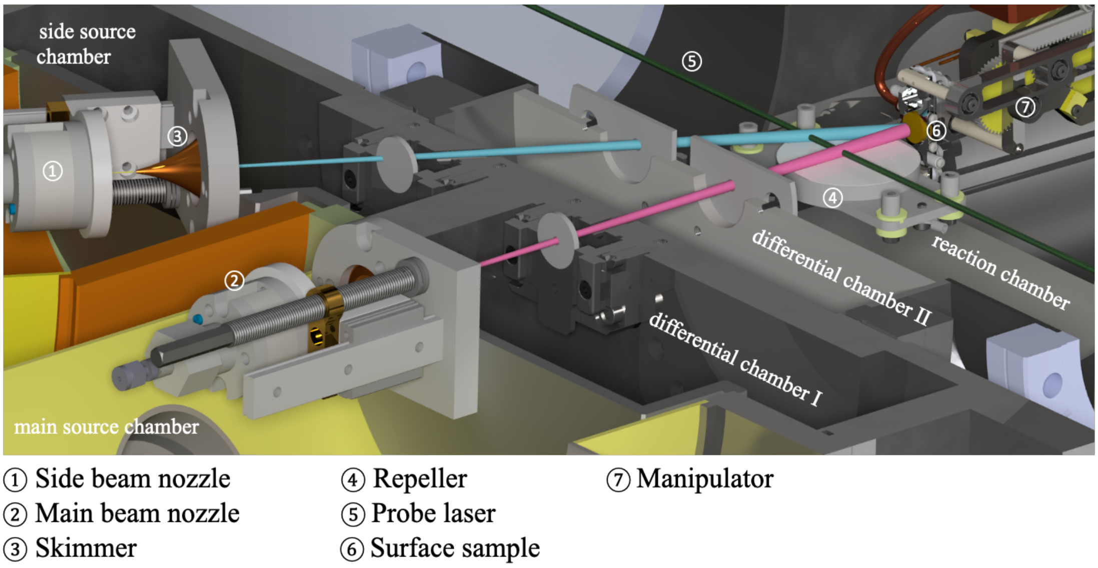

Figure S1. Schematic of the experimental setup of the dual molecular beam experiment apparatus with ultra-high-vacuum, which is composed of two source chambers and two differential chambers for the generation of the main beam and the side beam, respectively. The N_2O beam (blue line) and CO beam (red line) intersect at a Pd(110) surface in the reaction chamber.

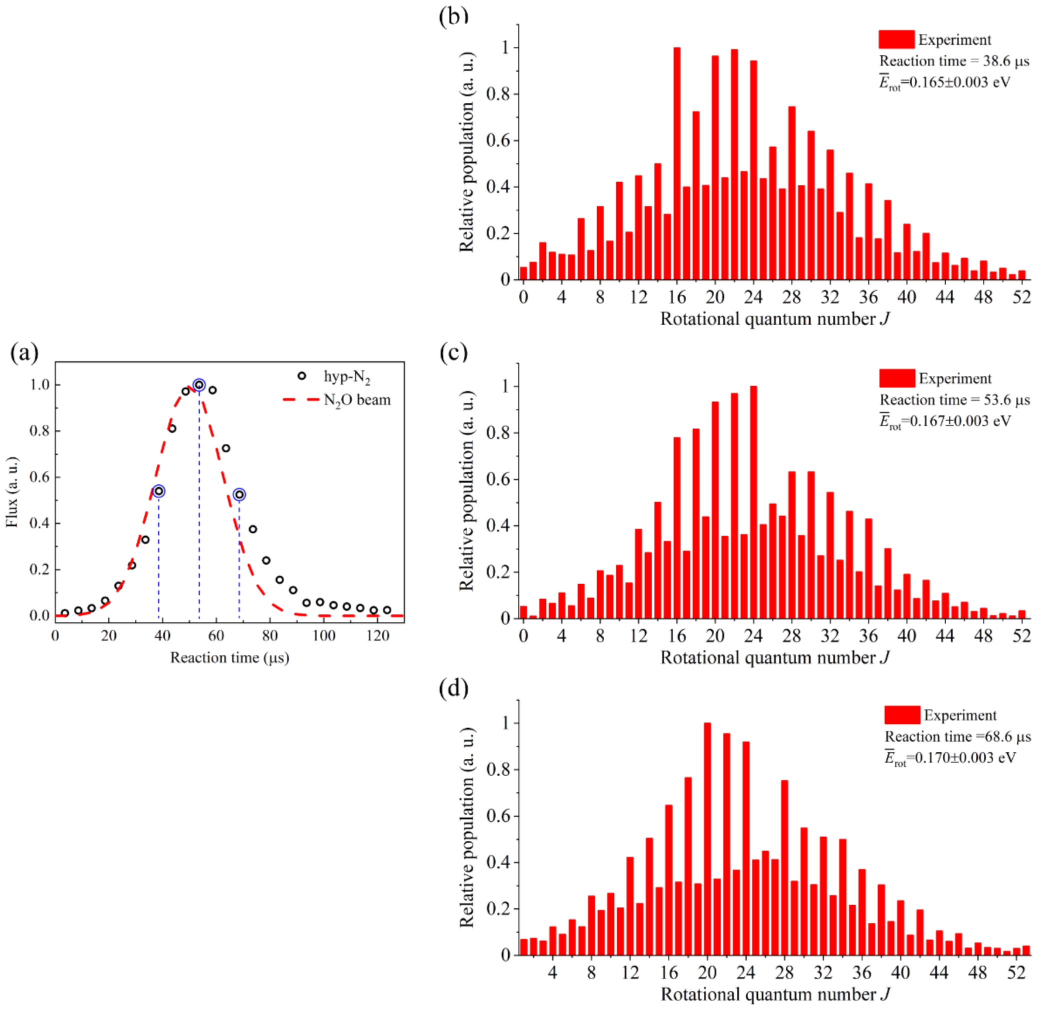

Figure S2. (2+1) REMPI spectra recorded for desorbing hyperthermal N_2 product at different delay times. (a) Reaction time distribution of the N_2 flux (open circles) compared to the incident N_2O beam. Reaction time-zero is defined as the arrival time of the N_2O beam at the surface. The surface temperature is 650 K and the O-coverage is 0.13 ML. Three vertical dashed lines represent the N_2 flux yielded at three different reaction times located at the period of rising, peaked, and decaying of N_2 signal, respectively. REMPI measurement was thus carried out to detect the N_2 flux generated at the three reaction times: 38.6, 53.6, and 68.6 μs, respectively. (b)–(d) show the J-state population distributions of N_2 at the three reaction times. The calculated $\overline{E}_{\mathrm{rot}}$ values are comparable from the different reaction times as 0.165 $\pm$ 0.003, 0.167 $\pm$ 0.003, and 0.170 $\pm$ 0.003 from the relative population distribution in (b), (c), (d), respectively.

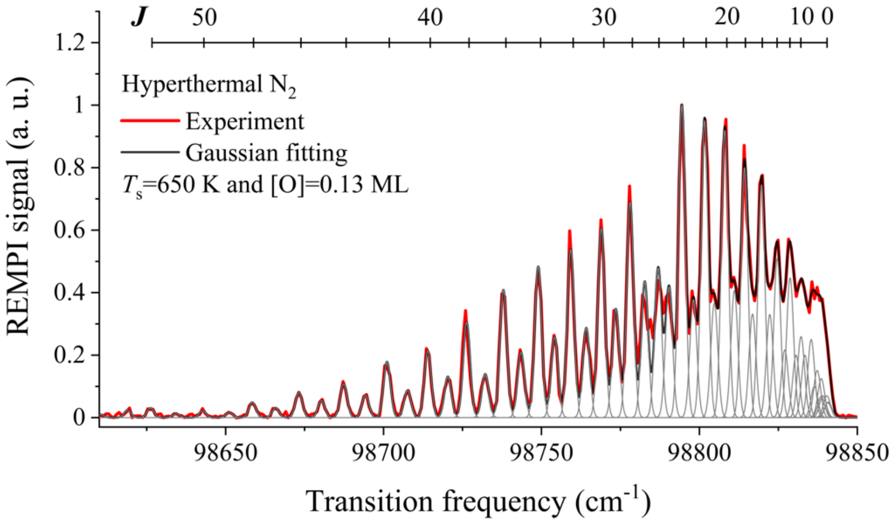

Figure S3. The peaks of (2+1) REMPI spectrum of hyperthermal N_2 is fit by multiple Gaussian functions with FHWM of 2.71 ± 0.024 cm^{-1}. The spectrum is the same as that in Fig. 3a. The combs indicate the rotational states labelled by *J*. The *x*-axis gives the wavenumber corresponding to the two-photon excitation energy.

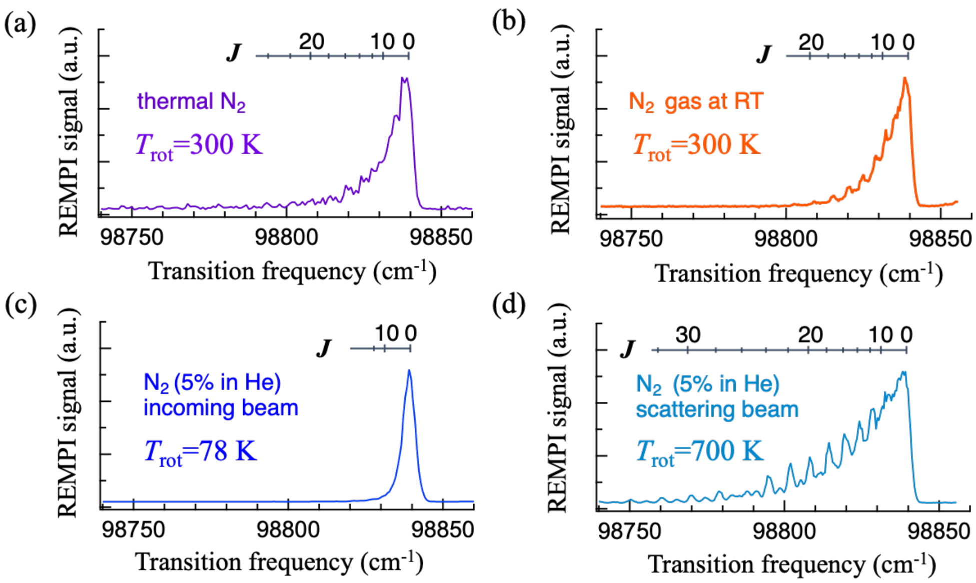

Figure S4. (2+1) REMPI spectra of N_2 obtained via the two-photon-resonant $a''\,{}^1\Sigma_g^+ \leftarrow X\,{}^1\Sigma_g^+$ (0, 0) transition arising from (a) N_2 thermal product in a velocity range of 400–800 m/s from N_2O reduction on Pd(110) at T_{surf}=650 K. The O-coverage is 0.13 ML. (b) N_2 gas leaking into the reaction chamber at room temperature; (c) N_2 incoming beam diluted in helium (5% of N_2 in He); (d) Scattering N_2 from the beam of 5% of N_2 in He from the Pd(110) at T_{surf}=650 K. The combs indicate the rotational states labelled by *J*. The *x*-axis gives the wavenumber corresponding to the two-photon excitation energy.

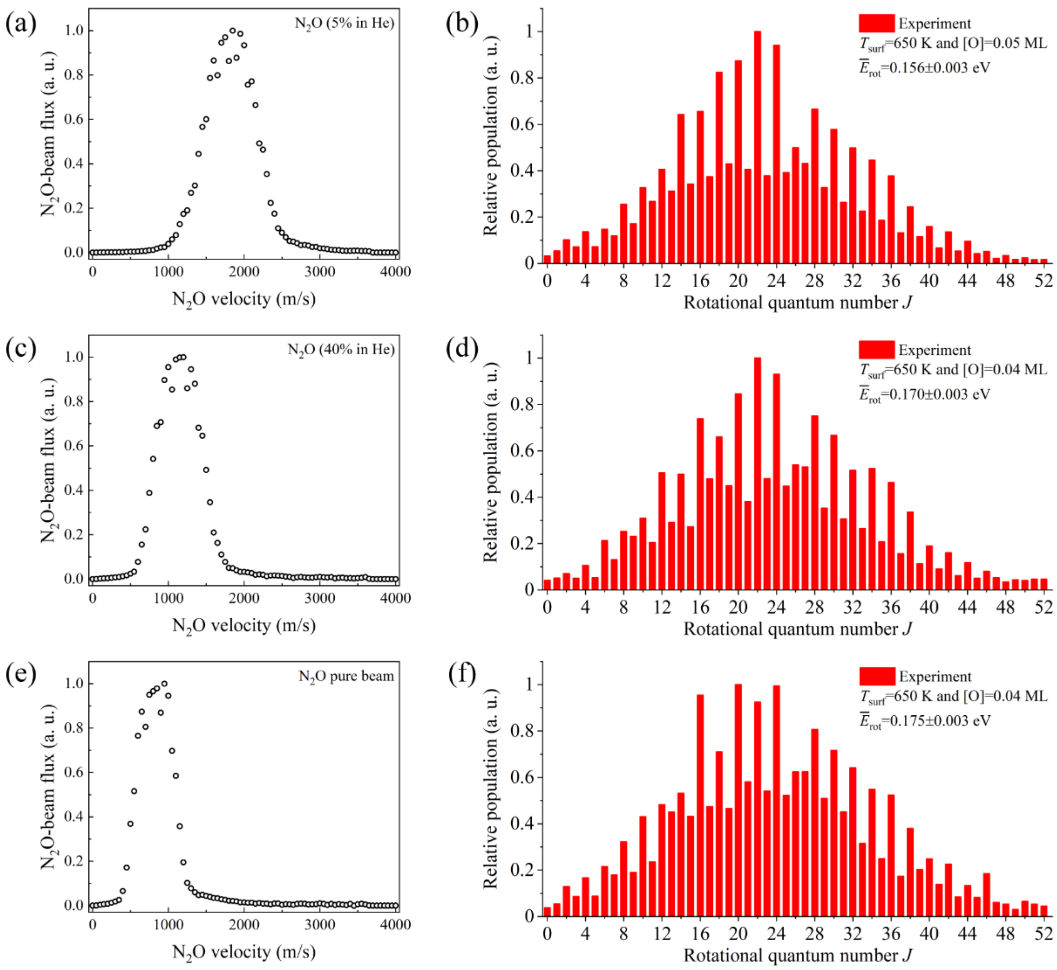

Figure S5. The rotational state distributions of hyperthermal N_2 product dependence on the N_2O incidence kinetic energies. (a) Velocity distribution of N_2O mixture beam (5% diluted in He) with an average translational energy of 0.46 eV. (c) Velocity distribution of N_2O mixture beam (40% diluted in He) with an average translational energy of 0.2 eV. (e) Velocity distribution of pure N_2O beam with an average translational energy of 0.11 eV. (b, d, f) Rotational state distributions of hyperthermal N_2 generated from the N_2O beam pulse prepared by a, c, and e, respectively. The T_{surf} is 650 K. The O-coverage is 0.05, 0.04, and 0.04 ML for (b), (d), and (f), respectively. The calculated $\bar{E}_{\mathrm{rot}}$ values are comparable as 0.156 ± 0.003, 0.170 ± 0.003, and 0.175 ± 0.003 from the relative distribution in (b), (d), and (f), respectively.

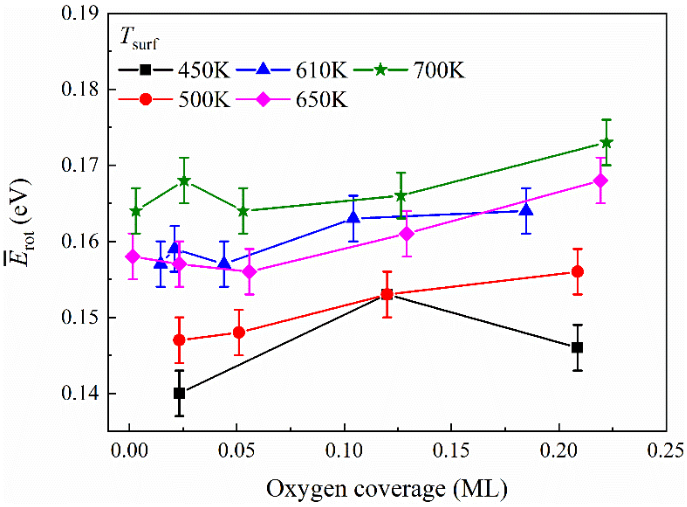

Figure S6. Effects of T_{surf} and O-coverage on the average rotational energy ($\bar{E}_{rot}$) of hyperthermal N_2 product. The saturation O-coverage on Pd(110) is 0.5 ML.[26]

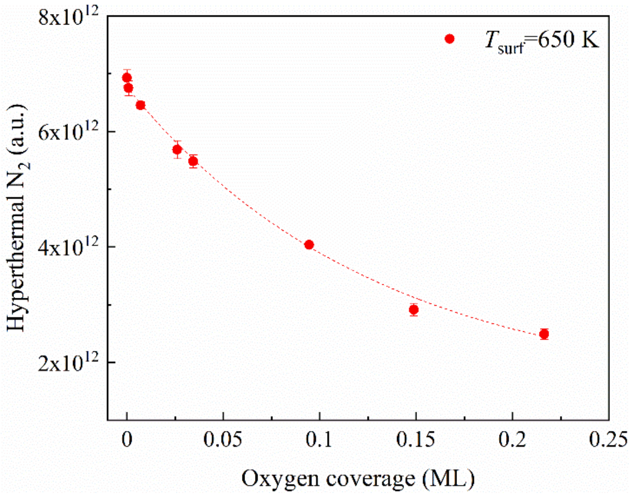

Figure S7. Effects of O-coverage on the flux of hyperthermal N_2 product at T_{surf} = 650 K. The dashed line is to guide the eye only. The saturation O-coverage on Pd(110) is 0.5 ML.[26]

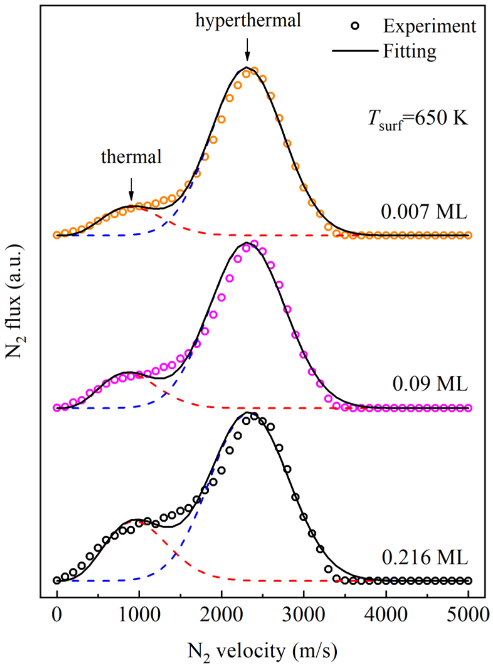

Figure S8. Velocity distributions of N_2 product from N_2O decomposition on Pd(110) with different O-coverages at T_{surf}=650 K. The N_2 flux was obtained by the signal in the ion images along the angles ranging from 40° to 50° with respect to the surface normal in the imaging plane.

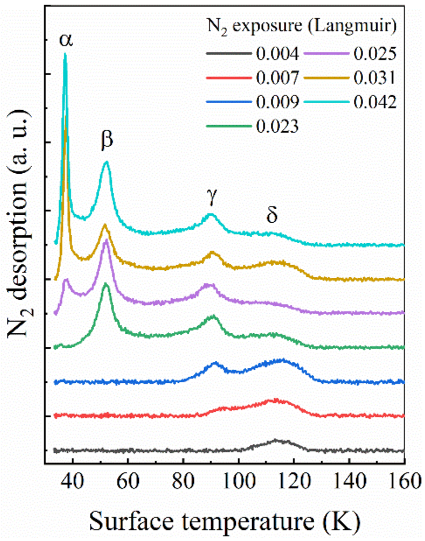

α peak (T_{max}= 37 ± 1 K)

A_{pre} (s^{-1})	E_{ad} (eV)
10^{12}	0.087
10^{13}	0.095
10^{14}	0.102

β peak (T_{max}= 52 ± 1 K)

A_{pre} (s^{-1})	E_{ad} (eV)
10^{12}	0.124
10^{13}	0.135
10^{14}	0.145

γ peak (T_{max}= 90 ± 2 K)

A_{pre} (s^{-1})	E_{ad} (eV)
10^{12}	0.220
10^{13}	0.237
10^{14}	0.255

δ peak (T_{max}= 113 ± 3 K)

A_{pre} (s^{-1})	E_{ad} (eV)
10^{12}	0.278
10^{13}	0.300
10^{14}	0.323

Figure S9. TPD profiles of N_2 (m/z=28) and Arrhenius information of the corresponding peaks. The Pd(110) surface is exposed to N_2 gas with various exposures from 0.004 to 0.042 L. The surface was held at the temperature of 34 K during the N_2 gas dosing. A_{pre} and E_{ad} represent the pre-exponential factor and the adsorption energy, respectively.

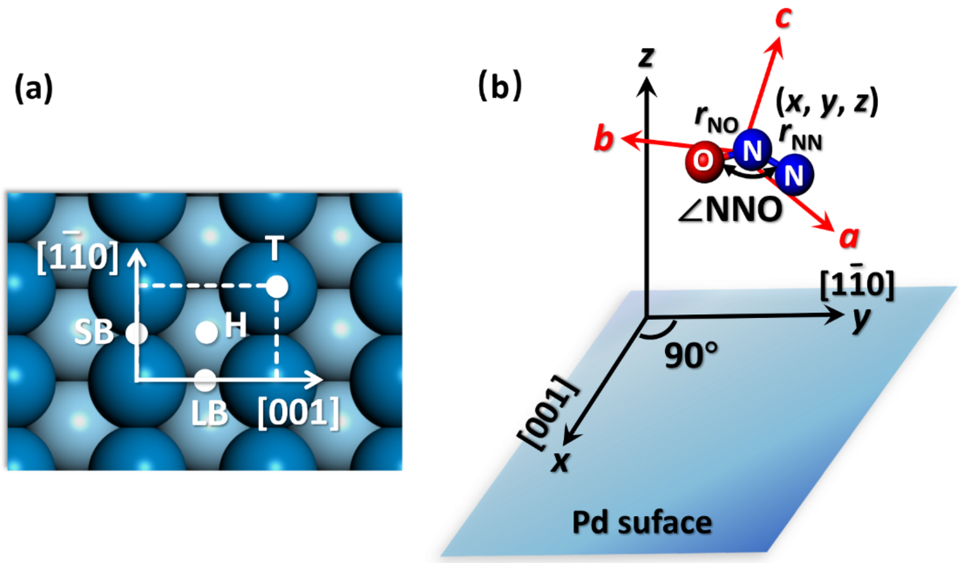

Figure S10. (a) Depiction of Pd(110) surface and the symmetry sites used in the system. T: top site, SB: short bridge site, LB: long bridge site, H: hollow site. (b) Coordinates for describing N_2O on the Pd(110) surface. The rotations of N_2O on the Pd(110) surface are described by the well-known three Euler angles (α, β, γ), between the Cartesian and body-fixed coordinate systems. The body-fixed coordinate system is defined by the *a, b, c* axes (red).

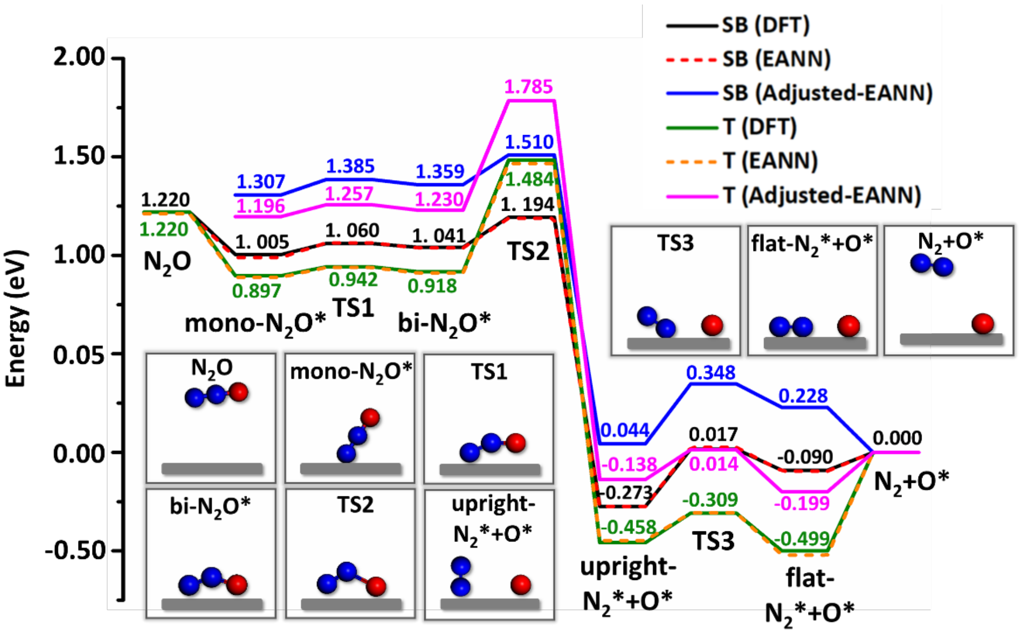

Figure S11. Comparison of geometries and energies (in eV) of adsorption states, transition states and decomposition products along the minimum energy paths of N_2O decomposition on the relaxed Pd(110) surface along [001] direction on short bridge (SB) and top (T) sites optimized directly with DFT, the EANN PES, and adjusted-EANN PES.

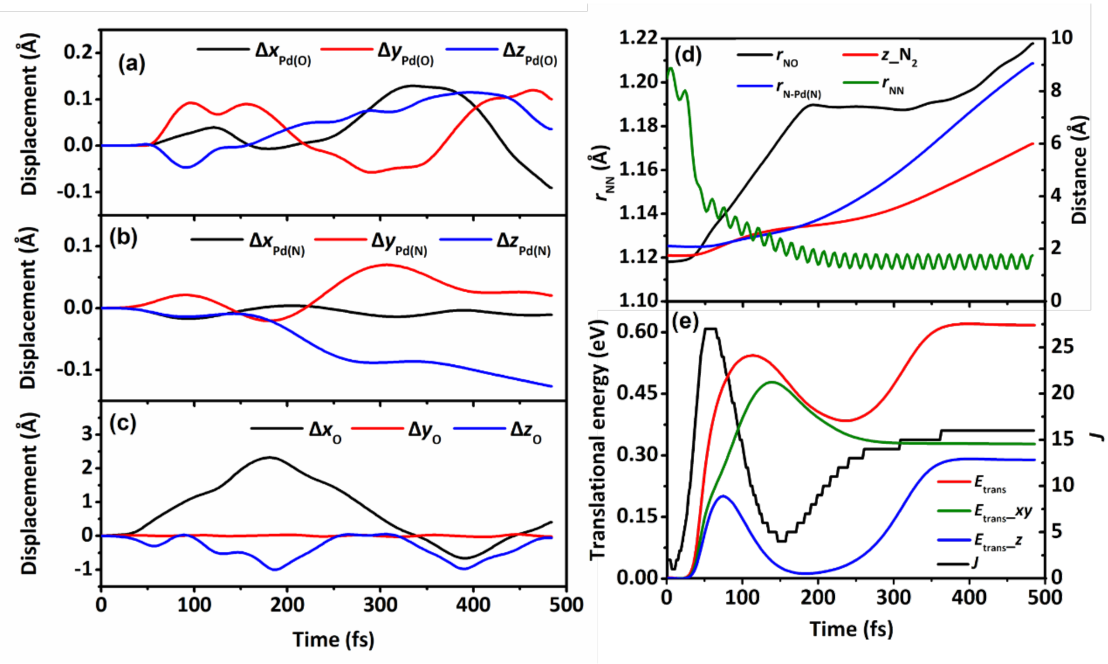

Figure S12. Evolution of geometry, kinetic energy, and rotational quantum number J as a function of time for a representation trajectory in the N_2O decomposition process from the transition state (Time=0 fs) at the short bridge site with initial kinetic energy roughly at 0 eV and T_{surf} of 0 K. The Pd(O) and Pd(N) represent the closest Pd atoms to the O and N atoms, respectively. Δx_i, Δy_i and Δz_i, i=Pd(O), Pd(N), O represent the displacements of ith atom along the x, y, z directions (see Fig. S10) relative to the transition state, respectively.

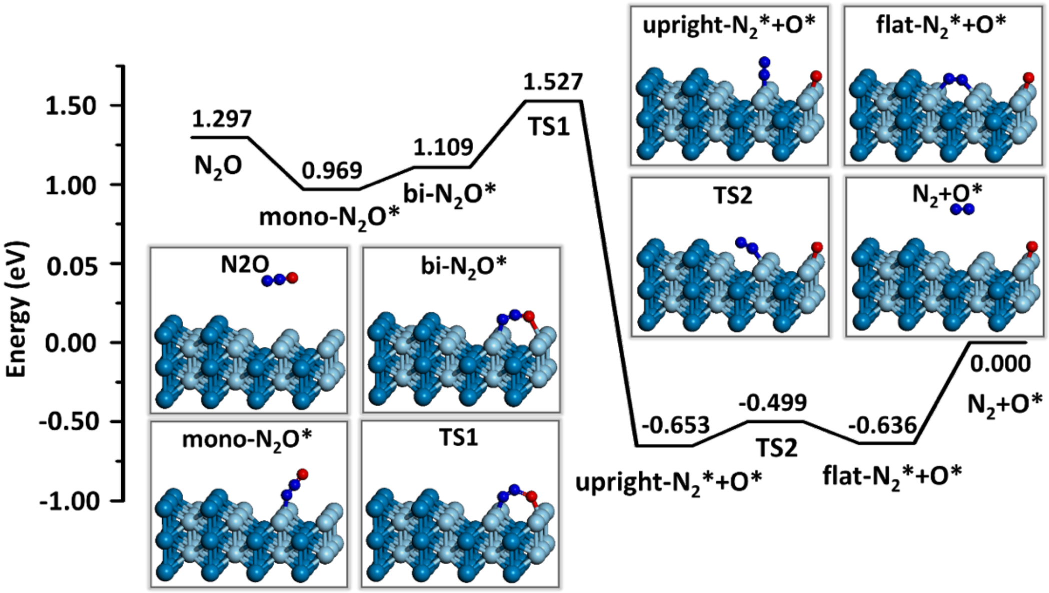

Figure S13. Potential energy diagram of the N_2O decomposition process at a defect site of Pd(110) surface with two Pd rows missing.

Table S1. Comparison of the DFT and PES molecular configurations (r_{NN}, r_{NO}, z, z_{N2}) for the adsorption state, transition state and decomposition products along the minimum energy path of N_2O* decomposition on the Pd(110) surface at short bridge (SB) site.

SB	DFT					EANN PES				
	r_{NN}(Å)	r_{NO}(Å)	∠NNO(°)	z (Å)	z_{N2} (Å)	r_{NN}(Å)	r_{NO}(Å)	∠NNO(°)	z (Å)	z_{N2} (Å)
N_2O	1.146	1.200	180.0	6.721	6.712	1.146	1.200	180.0	6.720	6.711
mono-N_2O*	1.162	1.203	177.5	2.843	2.313	1.162	1.205	178.6	2.953	2.346
TS1	1.181	1.228	154.8	2.051	1.953	1.177	1.223	157.8	2.097	1.974
bi-N_2O*	1.202	1.270	141.6	1.823	1.82	1.200	1.260	143.4	1.903	1.853
TS2	1.202	1.507	124.3	1.623	1.746	1.202	1.507	124.3	1.624	1.749
upright-N_2* + O*	1.140	3.602	54.8	1.666	2.279	1.140	3.651	55.9	1.660	2.262
TS3	1.158	5.230	1.6	1.178	1.520	1.160	5.230	2.4	1.162	1.489
flat-N_2* + O*	1.181	5.540	10.3	1.013	1.260	1.182	5.564	12.2	1.005	1.247
N_2+O*	1.117	-	-	-	8.927	1.117	-	-	-	8.930

S4. REFERENCES

(1) Zhao, Z.; Wang, Y.; Yang, X.; Quan, J.; Krüger, B. C.; Stoicescu, P.; Nieman, R.; Auerbach, D. J.; Wodtke, A. M.; Guo, H.; Park, G. B., Spin-Dependent Reactivity and Spin-Flipping Dynamics in O Atom Scattering from Graphite. *Nat. Chem.* **2023,** https://doi.org/10.1038/s41557-023-01204-2.

(2) Even, U.; Jortner, J.; Noy, D.; Lavie, N.; Cossart-Magos, C., Cooling of Large Molecules Below 1 K and He Clusters Formation. *J. Chem. Phys.* **2000,** *112*, 8068–8071.

(3) Neugebohren, J.; Borodin, D.; Hahn, H. W.; Altschäffel, J.; Kandratsenka, A.; Auerbach, D. J.; Campbell, C. T.; Schwarzer, D.; Harding, D. J.; Wodtke, A. M.; Kitsopoulos, T. N., Velocity-Resolved Kinetics of Site-Specific Carbon Monoxide Oxidation on Platinum Surfaces. *Nature* **2018,** *558*, 280–283.

(4) Borodin, D.; Golibrzuch, K.; Schwarzer, M.; Fingerhut, J.; Skoulatakis, G.; Schwarzer, D.; Seelemann, T.; Kitsopoulos, T.; Wodtke, A. M., Measuring Transient Reaction Rates from Nonstationary Catalysts. *ACS Catal.* **2020,** *10*, 14056–14066.

(5) Stoicheff, B. P., High Resolution Raman Spectroscopy of Gases: III. Raman Spectrum of Nitrogen. *Can. J. Phys.* **1954,** *32*, 630–634.

(6) Ma, Y.; Han, S.; Matsushima, T., Kinetics and Dynamics of N_2 Formation in a Steady-State N_2O + CO Reaction on Pd(110). *Langmuir* **2005,** *21*, 9529–9536.

(7) Hideyuki Horino; Suwen Liu; Atsuko Hiratsuka; Yuichi Ohno; Matsushima, T., Two-directional N_2 Desorption in Thermal Dissociation of N_2O on Pd(110) at Low Temperatures. *Chem. Phys. Lett.* **2001,** *341*, 419–424.

(8) Kresse, G.; Furthmüller, J., Efficient Iterative Schemes for ab initio Total-Energy Calculations Using a Plane-Wave Basis Set. *Phys. Rev. B* **1996,** *54*, 11169–11186.

(9) Kresse, G.; Furthmüller, J., Efficiency of ab-initio Total Energy Calculations for Metals and Semiconductors Ssing a Plane-Wave Basis Set. *Comp. Mater. Sci.* **1996,** *6* (1), 15–50.

(10) Perdew, J. P.; Burke, K.; Ernzerhof, M., Generalized Gradient Approximation Made Simple. *Phys. Rev. Lett.* **1996,** *77*, 3865–3868.

(11) Blöchl, P. E., Projector Augmented-Wave Method. *Phys. Rev. B* **1994,** *50*, 17953–17979.

(12) Henkelman, G.; Uberuaga, B. P.; Jónsson, H., A Climbing Image Nudged Elastic Band Method for Finding Saddle Points and Minimum Energy Paths. *J. Chem. Phys.* **2000,** *113*, 9901–9904.

(13) Henkelman, G.; Jónsson, H., A Dimer Method for Finding Saddle Points on High Dimensional Potential Surfaces using Only First Derivatives. *J. Chem. Phys.* **1999,** *111* (15), 7010–7022.

(14) Yin, R.; Jiang, B.; Guo, H., Mechanism and Dynamics of CO_2 Formation in Formic Acid Decomposition on Pt Surfaces. *ACS Catal.* **2022,** *12*, 6486–6494.

(15) Jiang, B.; Li, J.; Guo, H., Potential Energy Surfaces from High Fidelity Fitting of ab initio Points: the Permutation Invariant Polynomial-Neural Network Approach. *Int. Rev. Phys. Chem.* **2016,** *35*, 479–506.

(16) Behler, J., Perspective: Machine Learning Potentials for Atomistic Simulations. *J. Chem. Phys.* **2016,** *145*, 170901.

(17) Jiang, B.; Li, J.; Guo, H., High-Fidelity Potential Energy Surfaces for Gas-Phase and Gas–Surface Scattering Processes from Machine Learning. *J. Phys. Chem. Lett.* **2020,** *11*, 5120–5131.

(18) Jiang, B.; Guo, H., Dynamics in Reactions on Metal Surfaces: A Theoretical Perspective. *J. Chem. Phys.* **2019,** *150*, 180901.

(19) Zhang, Y.; Hu, C.; Jiang, B., Embedded Atom Neural Network Potentials: Efficient and Accurate Machine Learning with a Physically Inspired Representation. *J. Phys. Chem. Lett.* **2019,** *10*, 4962–4967.

(20) Behler, J., Atom-Centered Symmetry Functions for Constructing High-Dimensional Neural Network Potentials. *J. Chem. Phys.* **2011,** *134*, 074106.
(21) Zhou, X.; Zhang, Y.; Yin, R.; Hu, C.; Jiang, B., Neural Network Representations for Studying Gas-Surface Reaction Dynamics: Beyond the Born-Oppenheimer Static Surface Approximation†. *Chin. J. Chem.* **2021,** *39*, 2917–2930.
(22) Hase, W. L.; Duchovic, R. J.; Hu, X. Y.; Komornicki, A.; Lim, K. F.; Lu, D. h.; Peslherbe, G. H.; Swamy, K. N.; Linde, S. R. V.; Varandas, A. J. C.; Wang, H.; Wolf, R. J. In *VENUS96: A General Chemical Dynamics Computer Program*, 1996; p 671.
(23) Jiang, B.; Guo, H., Dynamics of Water Dissociative Chemisorption on Ni(111): Effects of Impact Sites and Incident Angles. *Phys. Rev. Lett.* **2015,** *114*, 166101.
(24) Gutzwiller, M. C., *Chaos in Classical and Quantum Mechanics*. Springer New York, NY: 1990; Vol. 1, p XIV, 432.
(25) Kokalj, A.; Kobal, I.; Matsushima, T., A DFT Study of the Structures of N_2O Adsorbed on the Pd(110) Surface. *J. Phys. Chem. B* **2003,** *107*, 2741–2747.
(26) Tanaka, H.; Yoshinobu, J.; Kawai, M., Oxygen-Induced Reconstruction of the Pd(110) Surface: an STM Study. *Surf. Sci.* **1995,** *327*, L505–L509.
